# Alzheimer’s disease and oral manifestations: a bi-directional Mendelian randomization study

**DOI:** 10.3389/fneur.2024.1391625

**Published:** 2024-05-15

**Authors:** Jingxuan Huang, Aiping Deng, Yunshuang Bai, Chunyu Li, Huifang Shang

**Affiliations:** ^1^Department of Neurology, Laboratory of Neurodegenerative Disorders, National Clinical Research Center for Geriatric, West China Hospital, Sichuan University, Chengdu, China; ^2^Department of Neurology, West China Hospital, Sichuan University/West China School of Nursing, Sichuan University, Chengdu, China; ^3^Outpatient Department, West China Hospital, Sichuan University, Chengdu, China

**Keywords:** Alzheimer’s disease, oral conditions, oral cavity cancer, Mendelian randomization, causation analysis

## Abstract

**Background:**

Epidemiological studies have provided evidence suggesting an association between Alzheimer’s disease (AD) and various oral manifestations. However, conflicting conclusions have been drawn, and whether a causal association truly exists remains unclear.

**Methods:**

In order to investigate the potential causal association between AD and prevalent oral diseases, we conducted a bi-directional two-sample Mendelian randomization analysis based on summary statistics from genome-wide association studies of AD (*N* = 63,926), as well as mouth ulcer (*N* = 461,103), oral cavity cancer (*N* = 4,151), and periodontal disease (*N* = 527,652).

**Results:**

We identified that one standard increase in the risk of AD was causally associated with a reduced risk of oral cavity cancer (OR = 0.76, 95% CI: 0.63–0.92, *p* = 3.73 × 10^−3^). In the opposite direction, oral conditions were not causally associated with risk of AD.

**Conclusion:**

The present findings contributed to a better understanding of the correlation between AD and oral conditions, specifically oral cavity cancer. These results also identified new avenues for exploring the underlying mechanisms of oral cavity cancer.

## Introduction

The world’s older population is continuing to grow at an unprecedented rate. Around 8.5% of people worldwide are aged 65 and over nowadays, and this percentage is projected to nearly double over the next three decades. Oral health has been a neglected dimension of global health in the aging population (1). For example, oral cavity cancer is of particular concern for older adults, with around 70% diagnosed between the ages of 46 and 75 years old (2). The prevalence of periodontal disease increased gradually with age (3), and is highest in those aged 60 years and older (4). Mouth ulcers affect individuals of all ages, but are more common in older adults (5). Oral diseases and conditions that are associated with aging concomitantly result in an increased need for preventive, restorative, and periodontal dental care.

Alzheimer’s disease (AD) is the most common manifestation of elderly dementia, characterized by extracellular β-amyloid peptide, hyperphosphorylated tau protein, and neuronal or synaptic loss. Close correlation has been identified between AD and common oral conditions (6, 7). For example, compared to older people who are cognitively intact, older people who develop dementia are at increased risk of establishing oral health problems, such as periodontitis and ulcers (8). Among those aged 65 years and over, AD incidence and mortality were consistently associated with periodontal disease (9), and previous study has shown that older people with periodontal disease may have an increased risk of AD (10). Meanwhile, inverse association has been identified between AD and various types of cancer, suggesting potential shared inverse etiological mechanisms (11). A previous longitudinal population-based cohort study identified that patients with oral cavity cancer were in higher risk of developing AD (HR = 1.74, 95% CI: 1.27–2.83) (12), though negative result has also been reported (13). The precise mechanism and aetiology of oral manifestations in AD is not clear. One hypothesis is the chronic infection and system inflammation (14). In oral diseases, the exacerbated host inflammatory response is associated with greater amount of tissue damage, while inflammation plays a key role in the pathophysiology of AD. Another mechanism that has been suggested to underlie association between poor oral health and AD is diet and nutrition. Chewing deficiency among the elderly affects quality of diet and nutrient intake by leading to reliance on mashed food, carbohydrate, and confectioneries, as well as fewer fruits and vegetables, which might be risk factor of AD (15). In addition, AD patients have greater impairment of oral health because of their progressive cognitive impairment, which would affect their oral hygiene habits. Poor oral hygiene, difficulty in wearing dentures, and the inability to self-care such as carrying out oral hygiene procedures are the probable causes of impaired oral health in AD (6). These multiple lines of evidence suggested important correlation between AD and oral health, yet the complex interplay between AD and oral conditions complicates the analysis. Observational studies face the inherent challenge of accounting for confounding biases without the benefit of randomization, making it difficult to establish causation. Therefore, the question of whether AD plays a causal role in these oral manifestations remains largely unknown.

In this context, we aimed to explore the causal association between AD and prevalent oral diseases with two-sample Mendelian randomization (MR) analysis, which is a statistical method increasingly utilized to establish causal relationship between an exposure and a specific outcome. This method relies on leveraging single nucleotide polymorphisms (SNPs) as instrumental variables to strengthen the validity of causal inference (16, 17). By employing genetic variants as instrumental variables, MR offers a means to mitigate bias resulting from unobserved confounding between the exposure and outcome variables. In the current study, we investigated the causal relationship between AD and prevalent oral diseases, specifically mouth ulcers, periodontal disease, and oral cavity cancer, and identified that AD was associated with a decreased risk of oral cavity cancer.

## Methods

### GWAS summary datasets

The GWAS summary datasets utilized in the current study were provided in Supplementary Table S1. We obtained summary statistics of AD from a previous genome-wide association study (GWAS) among 21,982 cases and 41,944 controls of European ancestry (18). To select instrumental variables, we identified SNPs that met the genome-wide significance threshold (*p* < 5 × 10^−8^) and exhibited low linkage disequilibrium (*r*^2^ < 0.001) with other SNPs within a clumping distance of 10,000 kb as exposure for each trait. We analyzed three oral diseases as outcomes, including mouth ulcer (*N* = 461,103) (19), periodontal disease (*N* = 527,652) (20), and oral cavity cancer (*N* = 4,151) (21), based on available summary statistics from previous GWAS. The effects were harmonized to ensure that the effect estimates in both the exposure and outcome GWAS corresponded to the same allele for each SNP.

### Two-sample Mendelian randomization analysis

Conducting MR analysis requires three fundamental assumptions: (A) relevance assumption: the instrumental variables used are associated with the exposure of interest; (B) independence assumption: the instrumental variables are independent of any confounding factors that may influence the association between the exposure and the outcome; and (C) exclusion-restriction assumption: the instrumental variables are conditionally independent of the outcome, given the exposure and the confounding factors. These assumptions serve as prerequisites for valid causal inference in MR analyses.

In our study, a two-sample MR analysis was conducted using the random effects inverse variance weighted (IVW) method. This approach allowed us to assess the causal effect of AD on the risk of oral diseases. Bonferroni-corrected thresholds (0.05/3 = 0.017) were adopted to account for multiple testing. Additionally, to validate the significant findings, we employed the weighted median and weighted mode methods as alternative approaches. These two methods were used as supplementary analyses to confirm the robustness and consistency of the results.

In the second stage, we investigated whether oral conditions, acting as a potential risk factor, could causally influence the risk of AD. We conducted MR analysis to assess the causal role of oral conditions in AD with the same workflow. SNPs meeting the genome-wide significance threshold (*p* < 5 × 10^−8^) and having low linkage disequilibrium (*r*^2^ < 0.001) with other SNPs within a clumping distance of 10,000 kb were chosen as exposure for each oral disease, while summary statistics of AD were used as the outcome. Given that no loci were significant (*p* < 5 × 10^−8^) for oral cavity cancer and periodontal disease, a more relaxed significance threshold (*p* < 1 × 10^−5^) was used.

In order to assess the potential violation of MR assumptions in the analysis, we conducted several sensitivity analyses to evaluate the robustness of our results and to account for any potential biases or violations of the MR assumptions. To assess the strength of the effect of each SNP on the exposure traits, we calculated the F-statistic. SNPs with an F-statistic below 10 were considered as weak instruments and subsequently excluded from the analysis. It is important to address the issue of pleiotropy, where the selected SNPs may have effects on multiple traits. To identify and mitigate the influence of horizontal pleiotropy outliers, we employed MR-PRESSO, a statistical method specifically designed for detecting and correcting for such outliers. The identified outliers were removed from the analysis to minimize the impact of horizontal pleiotropy on the results. Cochran’s *Q* statistic, which is derived from the IVW estimate, should follow a *χ*^2^ distribution with degrees of freedom equal to the number of SNPs minus 1. In order to assess heterogeneity in the MR estimates, we conducted Cochran’s *Q* test, which allowed us to examine if there was significant variation across the instrumental variables in estimating the causal effect. Additionally, we employed MR-Egger regression, which is a weighted linear regression model that incorporates the SNP-outcome effects and SNP-exposure effects, allowing for the estimation of the intercept. The intercept in MR-Egger regression provides an estimate of the average pleiotropic bias, which can indicate the presence of potential pleiotropy in the analysis. The statistical power was calculated at http://cnsgenomics.com/shiny/mRnd/. The R package TwoSampleMR 0.5.6 was used for the statistical analyses.

## Results

**Figure 1:**
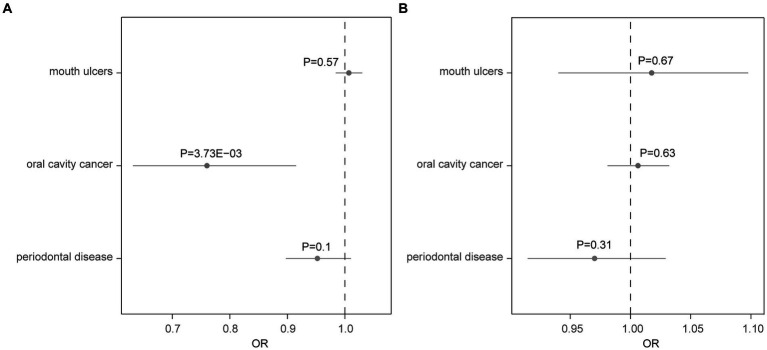
Forest plot showing results from the Mendelian randomization analysis. Results from the Mendelian randomization (MR) analysis to evaluate causal role of **(A)** Alzheimer’s disease in three oral conditions, and **(B)** three oral conditions in Alzheimer’s disease. Estimates are per 1 standard deviation (SD) increase in the trait. Bold *p*-value denotes significant association (*p* < 0.017).

**Figure 2:**
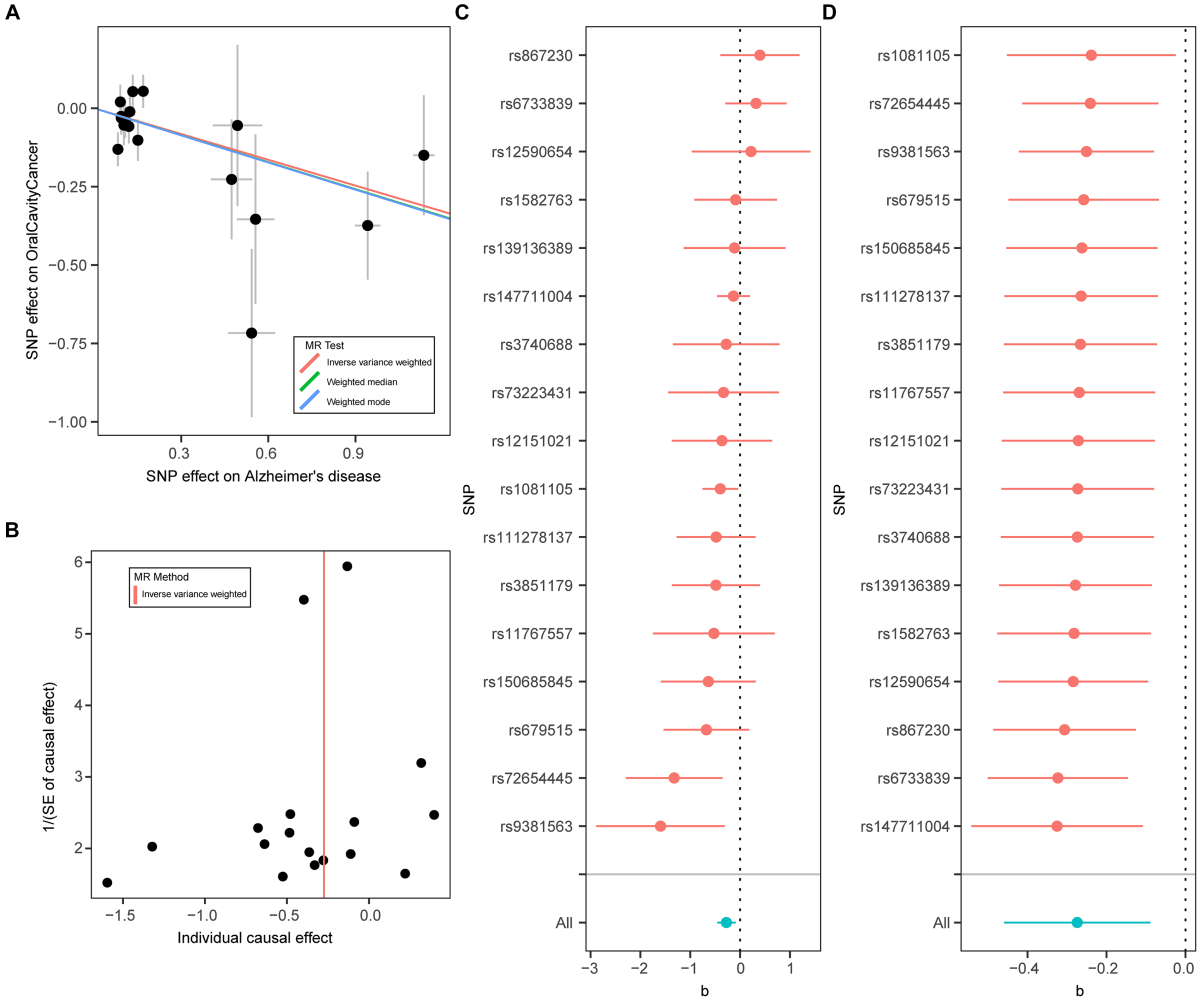
Mendelian randomization analysis results for Alzheimer’s disease on risk of oral cavity cancer. **(A)** Scatter plot of genetic associations with Alzheimer’s disease (horizontal lines) against genetic associations with oral cavity cancer (vertical lines). Error bars for genetic associations are 95% confidence intervals. The slopes of each line in the scatter plot represent the causal association for each method. **(B)** Funnel plot of single-SNP effect estimates and corresponding inverse standard errors. **(C)** Forest plot of the association of individual SNPs with Alzheimer’s disease and oral cavity cancer, together with pooled estimates. **(D)** Forest plot of the results of the leave-one-out sensitivity analysis, where each SNP in the instrument was iteratively removed from the instrumental variables.

Using the two-sample MR approach, we investigated the potential role of AD in the risk of three common oral diseases. Results showed that one standard deviation increase in genetically determined higher risk of AD was significantly associated with a reduced risk of oral cavity cancer (OR = 0.76, 95% CI: 0.63–0.92, *p* = 3.73 × 10^−3^) (Figure 1). Such association was further verified by the weighted median (OR = 0.75, 95% CI: 0.59–0.96, *p* = 0.023) and weighted mode (OR = 0.75, 95% CI: 0.58–0.97, *p* = 0.042) methods (Figure 2). The funnel plot demonstrated a visually symmetrical distribution, indicating the absence of significant directional pleiotropy that could influence the estimates (Figure 2). In contrast, our analysis did not reveal a significant association between AD and the risk of mouth ulcers or periodontal disease. In the opposite direction, we did not find any significant associations suggesting that oral conditions act as risk factors for AD (Figure 1; Supplementary Figures S2–S5).

Furthermore, we performed a number of sensitivity analyses to validate the causal association between AD and the oral diseases. The Cochran’s *Q* test indicated no significant heterogeneity among the instrumental variables, suggesting consistent estimates of the causal association between AD and the oral diseases (Table 1; Supplementary Table S2). The *F* statistics of all the instrumental variables were above 10 (ranging from 19 to 962), indicating that the selected instruments were sufficiently strong to provide reliable estimates. Furthermore, the MR-Egger regression analysis did not provide evidence of horizontal pleiotropy, as the intercept was not significantly deviated from zero. Moreover, the MR-PRESSO analysis did not detect any potential instrumental outliers, indicating that the instrumental variables used in the analysis were not influenced by pleiotropic effects. Overall, these results enhanced the validity and reliability of the MR analysis results.

**Table 1:** Heterogeneity and horizontal pleiotropy analyses of Alzheimer’s disease in three oral conditions.

Oral conditions	Heterogeneity			Horizontal pleiotropy			MR-PRESSO *p*-value
	IVW *Q*	IVW *Q* df	IVW *p*	Egger intercept	SE	*p*	
Mouth ulcers	26.07	17	0.07	−1.00 × 10^−3^	3.74 × 10^−3^	0.79	0.11
Oral cavity cancer	19.07	16	0.26	3.81 × 10^−3^	2.67 × 10^−2^	0.89	0.28
Periodontal disease	13.70	16	0.62	−1.38 × 10^−2^	8.22 × 10^−3^	0.12	0.64

IVW, inverse variance weighted; *Q*, Cochran’s *Q* test estimate; df, Cochran’s *Q* test degrees of freedom; SE, standard error.

## Discussion

Previous clinical and epidemiological studies have indicated a close relationship between AD and various oral diseases. However, the conclusions drawn from these studies have been inconsistent. Moreover, unmeasured confounding factors in clinical studies can introduce biases that are difficult to account for. In light of these challenges, we conducted a study utilizing the MR approach to examine the causal role of AD in the risk of three oral diseases. Additionally, we investigated the reverse relationship between these oral diseases and AD. Our analysis revealed a significant association between AD and a decreased risk of oral cavity cancer. These findings contribute to a better understanding of the involvement of AD in oral diseases and provide insights that can aid in the investigation of the pathogenesis of oral cavity cancer.

Cancer and AD are common diseases in the aging population. Previous epidemiological studies have identified an inverse association between AD and various types of cancer, suggesting potential shared inverse etiological mechanisms (11, 22). However, conflicting results have also been reported (23). In the current study, we identified that AD was associated with a decreased risk of oral cavity cancer using the MR approach. Though the precise aetiology of reduced risk of oral cavity cancer in AD remains unclear, the biological mechanisms involved in both diseases have been proposed, such as sustaining proliferative signaling, cell death, and grow suppressors (24). Additionally, processes related to cell growth and survival, as well as the production of specific molecules including the anti-stress response protein vimentin and the enzyme carbonic anhydrase, are all upregulated in cancer, while these processes and proteins are downregulated in AD (25). Another study found that the proteins p53 and PIN1 were implicated in both cancer and Alzheimer’s biomarkers (26). These accumulating evidence pointed towards potential shared pathogenesis between AD and oral cavity cancer. In the opposite direction, we did not identify association between oral cavity cancer and AD, suggesting oral cavity cancer might not causally influence the risk of AD from the genetic perspective. In contrast, one previous epidemiological study has identified higher risk of AD in patients with oral cavity cancer (12). However, the increased risk might be due to treatment for the cancer. A majority of patients with oral cavity cancer will receive radiation therapy, while radiation-related brain damage will be caused as the treatment site is adjacent to the brain when radiation therapy is administered. Consistently, one previous cohort study showed that patients with nasopharyngeal cancer treated with radiation therapy were more likely to develop dementia than normal controls (27). Nevertheless, the sample size of the GWAS on oral cavity cancer used in the current study was small, with no significant variants identified. The suggestively significant variants used as instrumental variables might limit the statistical power. Therefore, replication studies with larger sample sizes were still needed. Meanwhile, more in-depth studies are required to gain a comprehensive understanding of the role of AD in the development and progression of oral cavity cancer. Further investigations can explore the underlying mechanisms, such as common molecular pathways or genetic factors that may contribute to the observed association.

Periodontitis is a prevalent chronic inflammatory disease affecting the teeth-supporting tissues, ultimately leading to tooth-loss if not treated. Periodontitis is linked to several systemic conditions with underlying pathophysiological features related to chronic inflammation or altered immune system function such as rheumatoid arthritis and diabetes mellitus. There is also increasing evidence that inflammation plays a key role in the pathophysiology of AD (28). From the epidemiological perspective, one previous study found that periodontitis was associated with cognitive impairment among 2,355 participants aged 60 years or over (29). However, it is important to note that the limited statistical power of our study may have influenced the results. Given this limitation, further replication of our findings is warranted to draw more definitive conclusions regarding the association between AD and periodontal disease.

Previous study has shown that patients with AD had poorer oral health than the general population (7). Cognitive impairment in patients with AD may contribute to the worsening of oral hygiene thus aggravating oral infections (30). Meanwhile, AD could cause frequent dehydration, which leads to dry mouth from low saliva production. This puts patients with AD at higher risk of tooth decay, gum disease, and tooth loss if not treated properly. In the current study, we did not identify a causal association between AD and the risk of mouth ulcer. However, it is important to acknowledge that the proportion of variance explained by the instrumental variables used in our study for the exposures was relatively small (Supplementary Table S3). This limited the statistical power to detect associations with moderate effect sizes. Therefore, it is important for future research to consider larger sample sizes or the inclusion of additional instrumental variables to enhance the power and detect potential associations with greater precision. In addition, the study results primarily derive from cohorts of European individuals, necessitating caution in generalizing these findings to other ethnic populations. Future research endeavors should prioritize the inclusion of diverse ancestral cohorts to validate and extend the observed associations.

In conclusion, our study revealed a significant association between AD and a decreased risk of oral cavity cancer. These findings contribute to a better understanding of the relationship between AD and oral conditions. By shedding light on the connection between AD and oral cavity cancer, our findings offer valuable insights that can inform further research and clinical practices.

## Data availability statement

The original contributions presented in the study are included in the article/Supplementary material, further inquiries can be directed to the corresponding authors.

## Ethics statement

Ethical review and approval was not required for the study on human participants in accordance with the local legislation and institutional requirements. Written informed consent from the patients/participants or patients/participants’ legal guardian/next of kin was not required to participate in this study in accordance with the national legislation and the institutional requirements.

## Author contributions

JH: Conceptualization, Data curation, Formal analysis, Methodology, Writing – original draft. AD: Writing – review & editing. YB: Writing – review & editing. CL: Validation, Visualization, Writing – review & editing. HS: Project administration, Supervision, Writing – review & editing.

## References

[1] Patel J Wallace J Doshi M Gadanya M Ben Yahya I Roseman J. Oral health for healthy ageing. Lancet Healthy Longev. (2021) 2:e521–7. doi: 10.1016/S2666-7568(21)00142-2, PMID: 36098001

[2] van Dijk BA Brands MT Geurts SM Merkx MA Roodenburg JL. Trends in oral cavity cancer incidence, mortality, survival and treatment in the Netherlands. Int J Cancer. (2016) 139:574–83. doi: 10.1002/ijc.30107, PMID: 27038013

[3] Kassebaum NJ Bernabé E Dahiya M Bhandari B Murray CJ Marcenes W. Global burden of severe periodontitis in 1990–2010: a systematic review and meta-regression. J Dent Res. (2014) 93:1045–53. doi: 10.1177/0022034514552491, PMID: 25261053 PMC4293771

[4] Kassebaum NJ Bernabé E Dahiya M Bhandari B Murray CJL Marcenes W. Global burden of untreated caries: a systematic review and metaregression. J Dent Res. (2015) 94:650–8. doi: 10.1177/0022034515573272 25740856

[5] Shi L Wan K Tan M Yin G Ge M Rao X. Risk factors of recurrent aphthous ulceration among university students. Int J Clin Exp Med. (2015) 8:6218–23. PMID: 26131228 PMC4483853

[6] Mancini M Grappasonni I Scuri S Amenta F. Oral health in Alzheimer’s disease: a review. Curr Alzheimer Res. (2010) 7:368–73. doi: 10.2174/156720510791162359 20043813

[7] Ribeiro GR Costa JLR Bovi Ambrosano GM Rodrigues Garcia RCM. Oral health of the elderly with Alzheimer’s disease. Oral Surg Oral Med Oral Pathol Oral Radiol. (2012) 114:338–43. doi: 10.1016/j.oooo.2012.03.028 22862974

[8] Delwel S Binnekade TT Perez RSGM Hertogh CMPM Scherder EJA Lobbezoo F. Oral hygiene and oral health in older people with dementia: a comprehensive review with focus on oral soft tissues. Clin Oral Investig. (2018) 22:93–108. doi: 10.1007/s00784-017-2264-2, PMID: 29143189 PMC5748411

[9] Beydoun MA Beydoun HA Hossain S El-Hajj ZW Weiss J Zonderman AB. Clinical and bacterial markers of periodontitis and their association with incident all-cause and Alzheimer’s disease dementia in a large national survey. J Alzheimers Dis. (2020) 75:157–72. doi: 10.3233/jad-200064, PMID: 32280099 PMC11008556

[10] Dominy SS Lynch C Ermini F Benedyk M Marczyk A Konradi A. *Porphyromonas gingivalis* in Alzheimer’s disease brains: evidence for disease causation and treatment with small-molecule inhibitors. Sci Adv. (2019) 5:eaau3333. doi: 10.1126/sciadv.aau3333, PMID: 30746447 PMC6357742

[11] Ospina-Romero M Glymour MM Hayes-Larson E Mayeda ER Graff RE Brenowitz WD. Association between Alzheimer disease and cancer with evaluation of study biases: a systematic review and meta-analysis. JAMA Netw Open. (2020) 3:e2025515. doi: 10.1001/jamanetworkopen.2020.25515, PMID: 33185677 PMC7666424

[12] Lee IH Yu H Ha SS Yang HG Kim DK. Increased risk of Alzheimer’s disease in patients with head and neck cancer. Cancers. (2023) 15:15 (23). doi: 10.3390/cancers15235516, PMID: 38067220 PMC10705604

[13] Bowles EJA Walker RL Anderson ML Dublin S Crane PK Larson EB. Risk of Alzheimer’s disease or dementia following a cancer diagnosis. PLoS One. (2017) 12:e0179857. doi: 10.1371/journal.pone.0179857, PMID: 28632787 PMC5478144

[14] Teixeira FB Saito MT Matheus FC Prediger RD Yamada ES Maia CSF. Periodontitis and Alzheimer’s disease: a possible comorbidity between oral chronic inflammatory condition and neuroinflammation. Front Aging Neurosci. (2017) 9:327. doi: 10.3389/fnagi.2017.00327, PMID: 29085294 PMC5649154

[15] Morris MC Evans DA Bienias JL Tangney CC Bennett DA Aggarwal N. Dietary fats and the risk of incident Alzheimer disease. Arch Neurol. (2003) 60:194–200. doi: 10.1001/archneur.60.2.194 12580703

[16] Sanderson E Glymour MM Holmes MV Kang H Morrison J Munafò MR. Mendelian randomization. Nat Rev Methods Primers. (2022) 2:6. doi: 10.1038/s43586-021-00092-5, PMID: 37325194 PMC7614635

[17] Li C Liu J Lin J Shang H. Covid-19 and risk of neurodegenerative disorders: a Mendelian randomization study. Transl Psychiatry. (2022) 12:283. doi: 10.1038/s41398-022-02052-3, PMID: 35835752 PMC9281279

[18] Kunkle BW Grenier-Boley B Sims R Bis JC Damotte V Naj AC. Genetic meta-analysis of diagnosed Alzheimer’s disease identifies new risk loci and implicates Aβ, tau, immunity and lipid processing. Nat Genet. (2019) 51:414–30. doi: 10.1038/s41588-019-0358-2, PMID: 30820047 PMC6463297

[19] Dudding T Haworth S Lind PA Sathirapongsasuti JF Tung JY Mitchell R. Genome wide analysis for mouth ulcers identifies associations at immune regulatory loci. Nat Commun. (2019) 10:1052. doi: 10.1038/s41467-019-08923-6, PMID: 30837455 PMC6400940

[20] Sakaue S Kanai M Tanigawa Y Karjalainen J Kurki M Koshiba S. A cross-population atlas of genetic associations for 220 human phenotypes. Nat Genet. (2021) 53:1415–24. doi: 10.1038/s41588-021-00931-x, PMID: 34594039 PMC12208603

[21] Lesseur C Diergaarde B Olshan AF Wünsch-Filho V Ness AR Liu G. Genome-wide association analyses identify new susceptibility loci for oral cavity and pharyngeal cancer. Nat Genet. (2016) 48:1544–50. doi: 10.1038/ng.3685, PMID: 27749845 PMC5131845

[22] Ma LL Yu JT Wang HF Meng XF Tan CC Wang C. Association between cancer and Alzheimer’s disease: systematic review and meta-analysis. J Alzheimers Dis. (2014) 42:565–73. doi: 10.3233/jad-140168 24906231

[23] Frain L Swanson D Cho K Gagnon D Lu KP Betensky RA. Association of cancer and Alzheimer’s disease risk in a national cohort of veterans. Alzheimers Dement. (2017) 13:1364–70. doi: 10.1016/j.jalz.2017.04.012, PMID: 28711346 PMC5743228

[24] Nudelman KNH McDonald BC Lahiri DK Saykin AJ. Biological hallmarks of cancer in Alzheimer’s disease. Mol Neurobiol. (2019) 56:7173–87. doi: 10.1007/s12035-019-1591-5, PMID: 30993533 PMC6728183

[25] Shafi O. Inverse relationship between Alzheimer’s disease and cancer, and other factors contributing to Alzheimer’s disease: a systematic review. BMC Neurol. (2016) 16:236. doi: 10.1186/s12883-016-0765-2, PMID: 27875990 PMC5120447

[26] Lanni C Masi M Racchi M Govoni S. Cancer and Alzheimer’s disease inverse relationship: an age-associated diverging derailment of shared pathways. Mol Psychiatry. (2021) 26:280–95. doi: 10.1038/s41380-020-0760-2, PMID: 32382138

[27] Penn IW Chung CH Huang YC Chen MC Sun CA Yip PK. Increased risk of dementia in patients with nasopharyngeal cancer treated with radiation therapy: a nationwide population-based cohort study. Arch Gerontol Geriatr. (2021) 93:104303. doi: 10.1016/j.archger.2020.104303, PMID: 33302001

[28] Leng F Edison P. Neuroinflammation and microglial activation in Alzheimer disease: where do we go from here? Nat Rev Neurol. (2021) 17:157–72. doi: 10.1038/s41582-020-00435-y, PMID: 33318676

[29] Noble JM Borrell LN Papapanou PN Elkind MSV Scarmeas N Wright CB. Periodontitis is associated with cognitive impairment among older adults: analysis of NHANES-III. J Neurol Neurosurg Psychiatry. (2009) 80:1206–11. doi: 10.1136/jnnp.2009.174029, PMID: 19419981 PMC3073380

[30] Syrjälä AM Ylöstalo P Sulkava R Knuuttila M. Relationship between cognitive impairment and oral health: results of the health 2000 health examination survey in Finland. Acta Odontol Scand. (2007) 65:103–8. doi: 10.1080/00016350601083521, PMID: 17453428

