# Supplementary material for: Alzheimer’s disease and oral manifestations: a bi-directional Mendelian randomization study
Source: Front Neurol. 2024 May 15;15:1391625. doi: 10.3389/fneur.2024.1391625 (PMC11138153; doi:10.3389/fneur.2024.1391625)

**Supplementary Table 1. Summary data from all GWAS used in the current study.**

| **Trait** | **Cases** | **Controls** | **Ethnic** | **Number of SNPs** | **PMID** |
| --- | --- | --- | --- | --- | --- |
| Alzheimer’s disease | 21,982 | 41,944 | EUR | 10,528,610 | 30820047 |
| Mouth ulcer | 47,079 | 414,024 | EUR | 10,599,054 | 30837455 |
| Oral cavity cancer | 1,223 | 2,928 | EUR | 7,294,750 | 27749845 |
| Periodontal disease | 11,300 | 516,352 | EUR & EAS | 20,457,829 | 34594039 |

EUR, European; EAS, East Asian; SNP, Single nucleotide polymorphism; GWAS, Genome-wide association study; PMID, PubMed ID.

**Supplementary Table 2. Heterogeneity and horizontal pleiotropy analyses of three oral conditions in Alzheimer’s disease.**

| **Oral conditions** | **Heterogeneity** | | | **Horizontal pleiotropy** | | | **MR-PRESSO  P value** |
| --- | --- | --- | --- | --- | --- | --- | --- |
|  | **IVW Q** | **IVW Q df** | **IVW P** | **Egger intercept** | **SE** | **P** |  |
| Mouth ulcers | 38.89 | 32 | 0.19 | 3.98E-03 | 6.52E-03 | 0.55 | 0.21 |
| Oral cavity cancer | 17.12 | 21 | 0.70 | -1.54E-02 | 1.25E-02 | 0.23 | 0.67 |
| Periodontal disease | 11.53 | 18 | 0.87 | 1.01E-02 | 7.03E-03 | 0.17 | 0.87 |

IVW, Inverse variance weighted; Q, Cochran’s Q test estimate; df, Cochran’s Q test degrees of freedom; SE, Standard error.

**Supplementary Table 3. Effect sizes can be detected with the power of 0.8 given the sample size, proportion of cases and variance explained by instrumental variables.**

| **exposure trait** | **outcome trait** | **sample size of outcome GWAS** | **proportion of cases in outcome GWAS** | **variance explained by instrumental variables** | **effect size** |
| --- | --- | --- | --- | --- | --- |
| Alzheimer’s disease | Mouth ulcer | 461,103 | 0.102 | 0.039 | 0.07 |
|  | Oral cavity cancer | 4,151 | 0.295 | 0.035 | 0.46 |
|  | Periodontal disease | 527,652 | 0.021 | 0.037 | 0.13 |
| Mouth ulcer | Alzheimer’s disease | 63,926 | 0.344 | 7.65E-03 | 0.25 |
| Oral cavity cancer |  | 63,926 | 0.344 | 0.132 | 0.06 |
| Periodontal disease |  | 63,926 | 0.344 | 7.68E-04 | 0.73 |

**Supplementary Figure 1. Mendelian randomization analysis results for Alzheimer’s disease on risk of oral ulcer.**


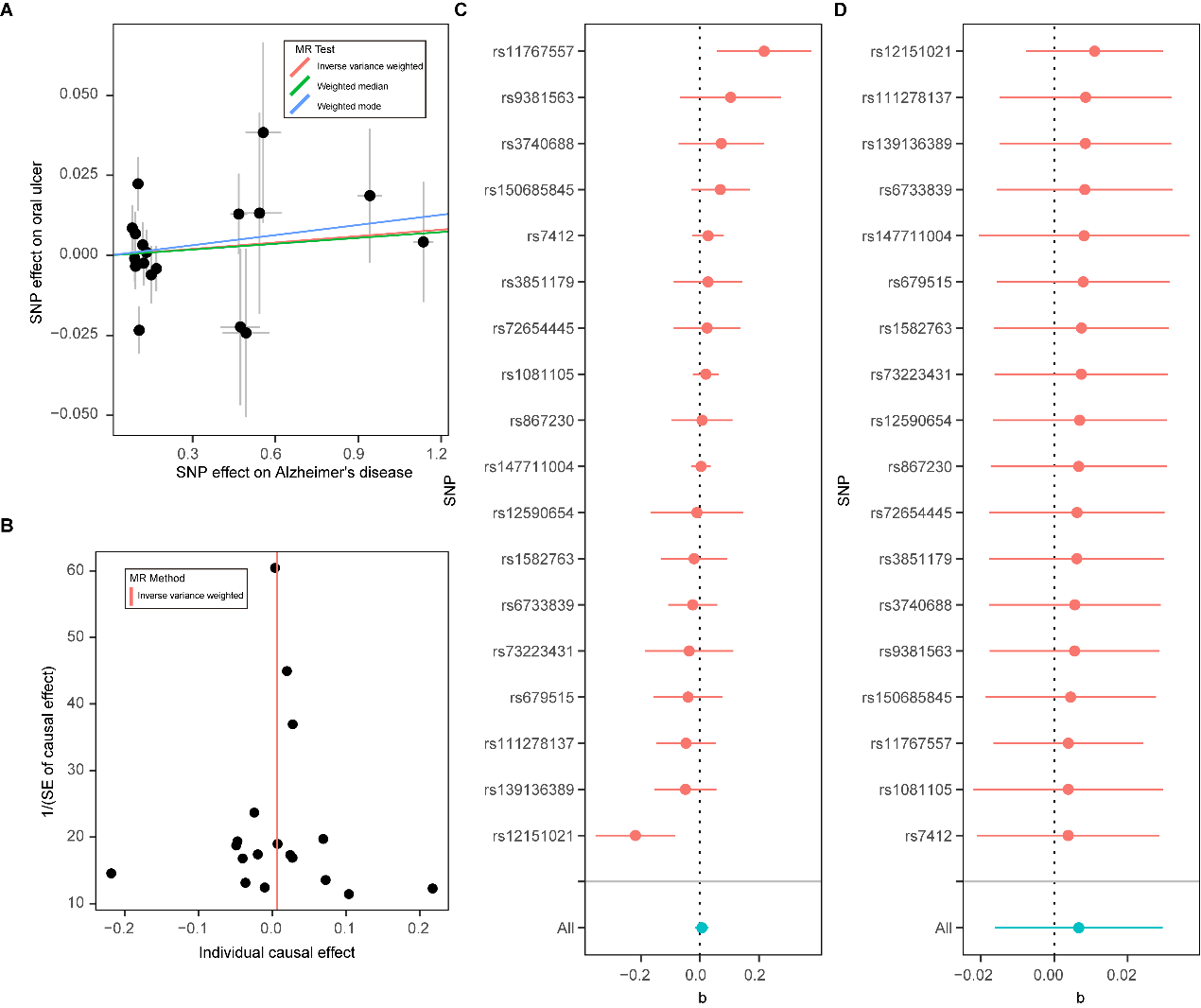


(**A**) Scatter plot of genetic associations with Alzheimer’s disease (horizontal lines) against genetic associations with oral ulcer (vertical lines). Error bars for genetic associations are 95% confidence intervals. The slopes of each line in the scatter plot represent the causal association for each method. (**B**) Funnel plot of single-SNP effect estimates and corresponding inverse standard errors. (**C**) Forest plot of the association of individual SNPs with Alzheimer’s disease and oral ulcer, together with pooled estimates. (**D**) Forest plot of the results of the leave-one-out sensitivity analysis, where each SNP in the instrument was iteratively removed from the instrumental variables.

**Supplementary Figure 2. Mendelian randomization analysis results for Alzheimer’s disease on risk of periodontal disease.**


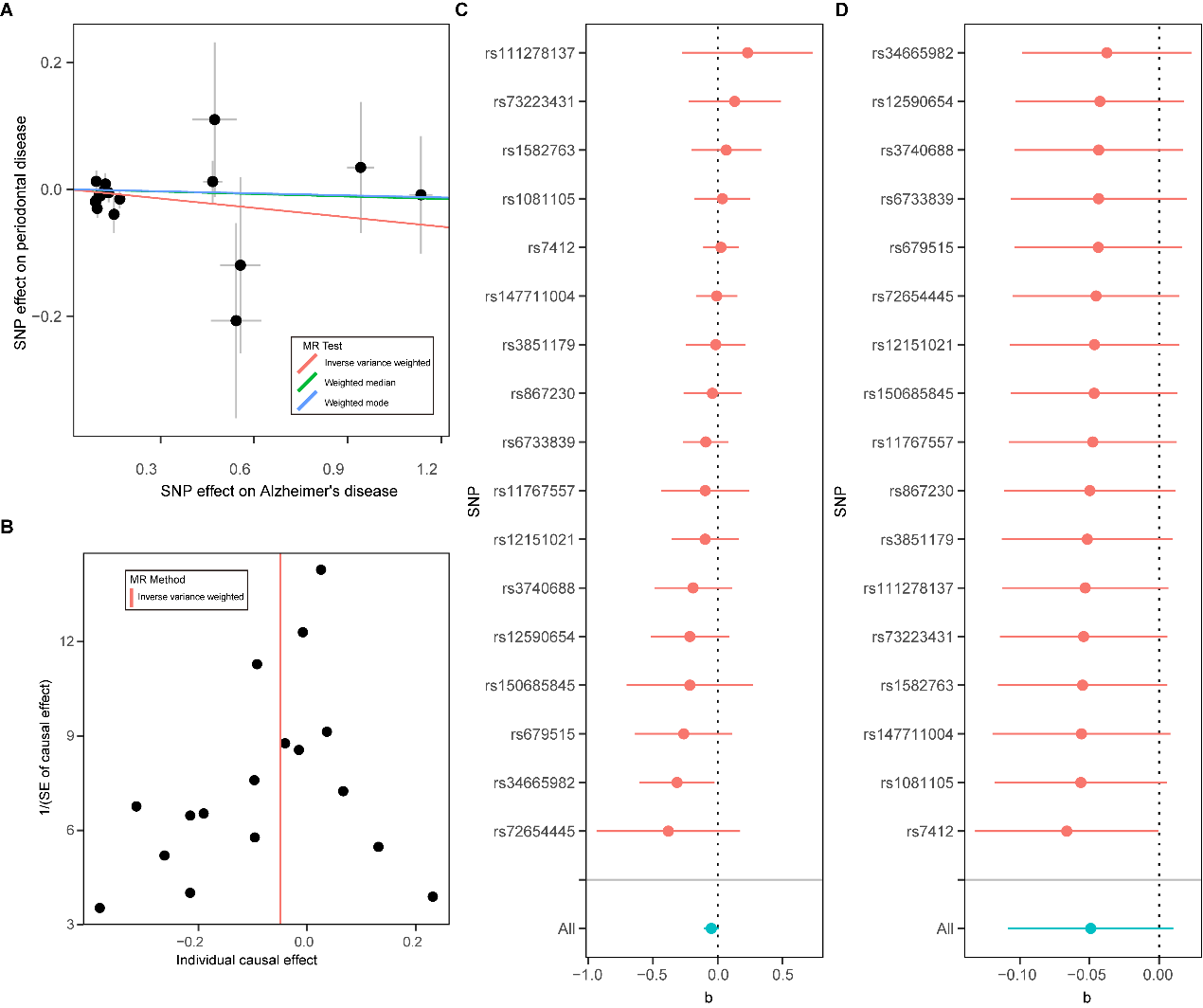


**Supplementary Figure 3. Mendelian randomization analysis results for oral cavity cancer on risk of Alzheimer’s disease.**


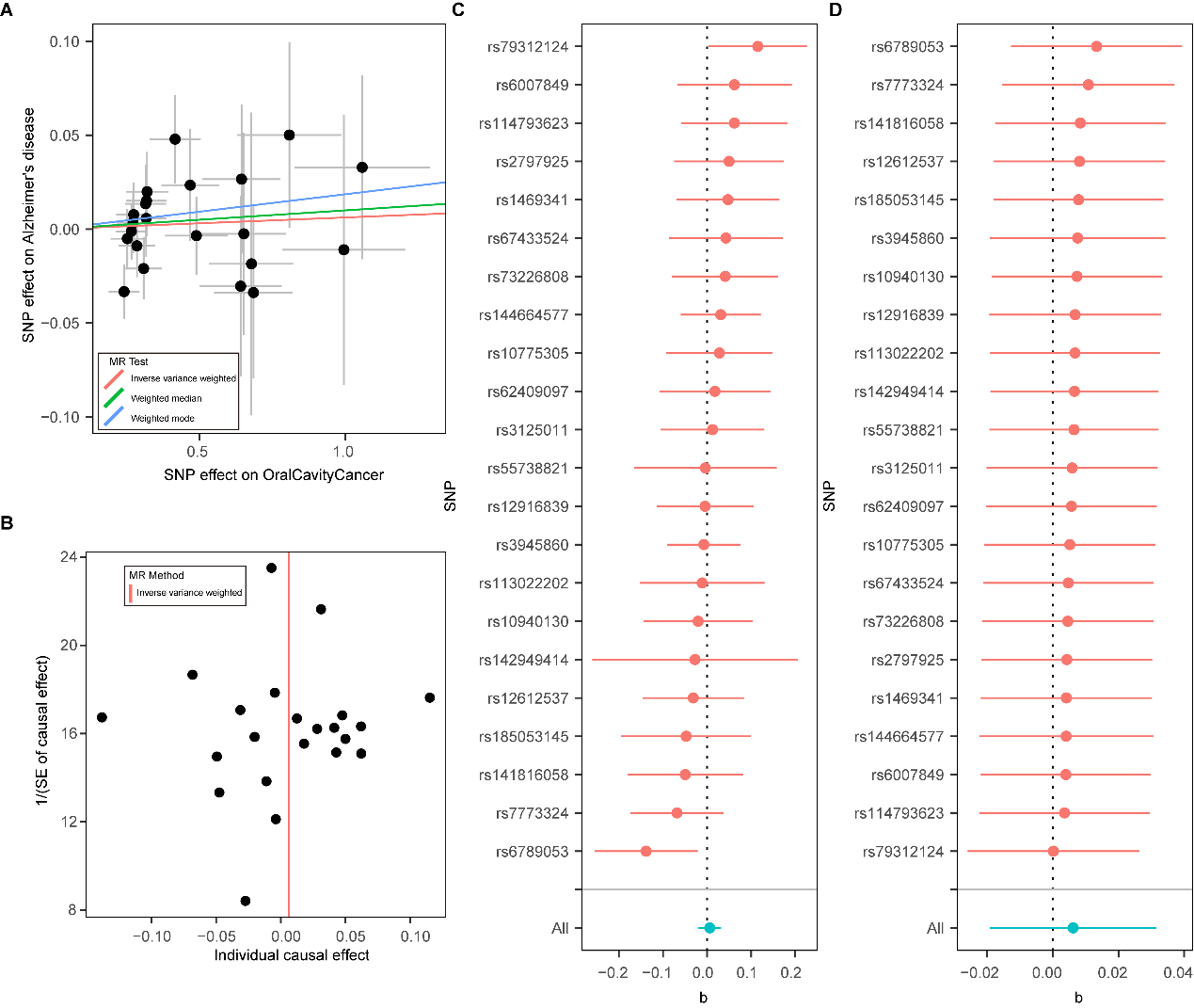


**Supplementary Figure 4. Mendelian randomization analysis results for oral ulcer on risk of Alzheimer’s disease.**


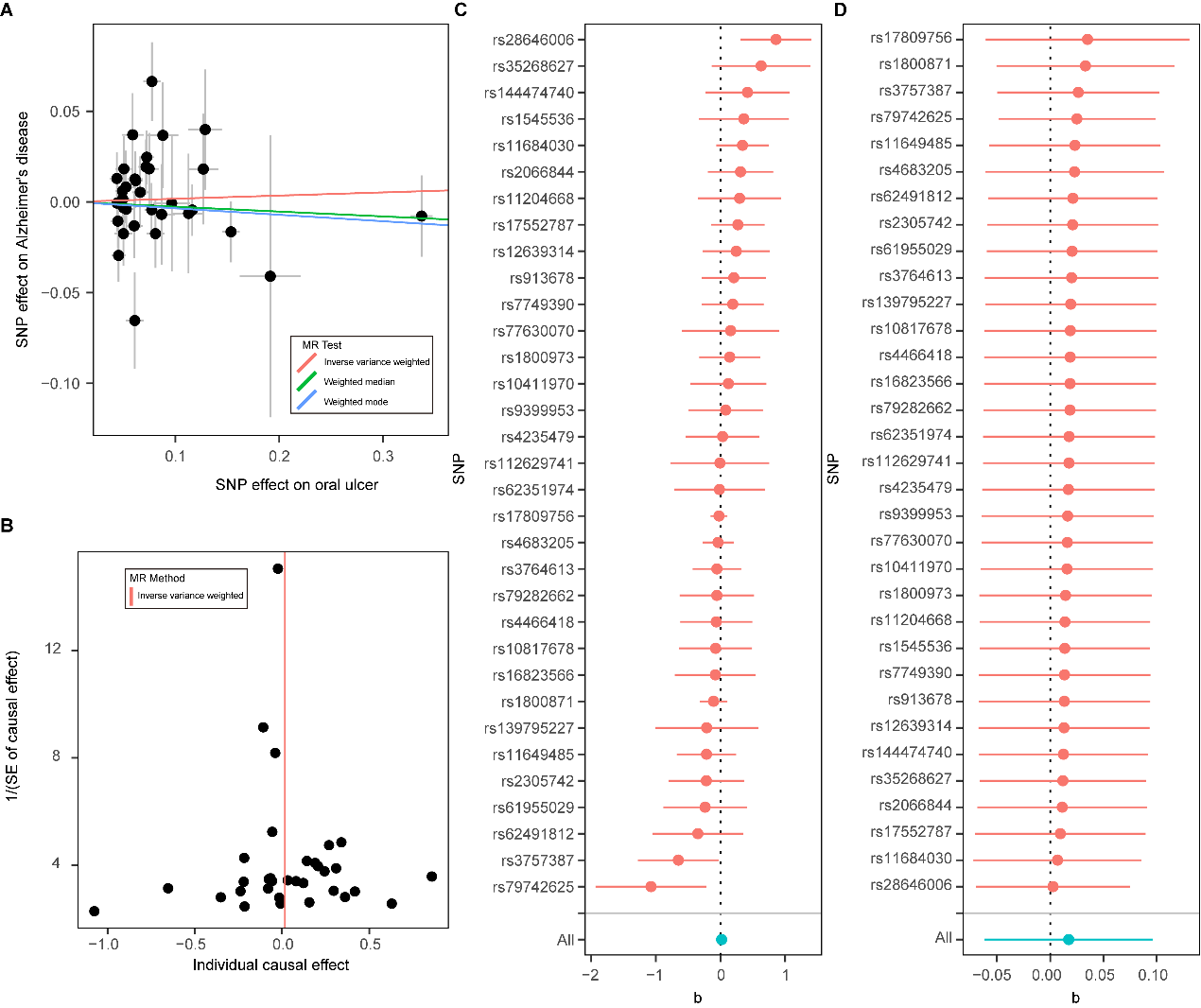


**Supplementary Figure 5. Mendelian randomization analysis results for periodontal disease on risk of Alzheimer’s disease.**


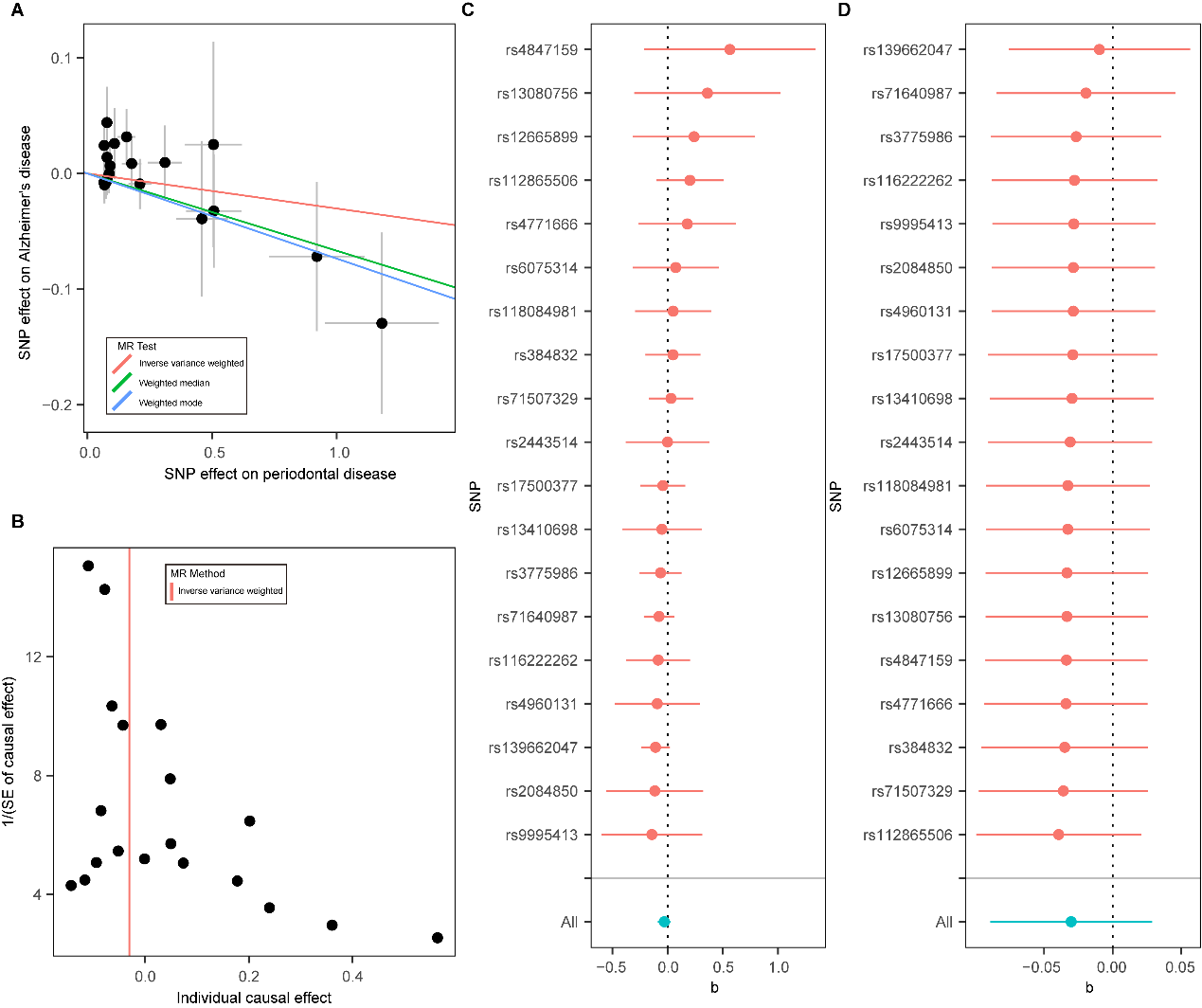

Supplement: Supplementary file 1 [file Data_Sheet_1.docx]
